# Desktop VR Is Better Than Non-ambulatory HMD VR for Spatial Learning

**DOI:** 10.3389/frobt.2019.00050

**Published:** 2019-07-09

**Authors:** Priyanka Srivastava, Anurag Rimzhim, Palash Vijay, Shruti Singh, Sushil Chandra

**Affiliations:** ^1^Perception and Cognition Group, Cognitive Science Lab, Kohli Research Centre on Intelligent Systems, International Institute of Information Technology-Hyderabad, Hyderabad, India; ^2^Department of Psychological Science, Central Connecticut State University, New Britain, CT, United States; ^3^Haskins Laboratories, New Haven, CT, United States; ^4^Indian Nuclear Medicine and Allied Sciences, Defence Research and Development Organization, New Delhi, India

**Keywords:** virtual reality, spatial knowledge, HMD, desktop, locomotion, immersion, motion-sickness, workload

## Abstract

Use of virtual reality (VR) technology is proliferating for designing and upgrading entertainment devices, and creating virtual environments that could be used for research and training. VR is becoming a strong research tool by providing a tighter control on the experimental environment and by allowing almost limitless possibilities of creating ecologically valid stimuli. However, the enhanced fidelity between the real and virtual worlds that VR provides does not always benefit human performance. For a better understanding, and increasing VR's usability, we need to identify the relevant constituent components of immersive technologies, and differentiate their roles, for example, how visual and interaction fidelity differentially improves human performance. We conducted an experiment to examine how two common VR display modes, head mounted display (HMD) and desktop (DT), would affect spatial learning when we restrict ambulatory locomotion in HMD. This manipulation allowed examining the role of varying visual fidelity with low interaction fidelity. We used a between-group design with 40 naïve participants. They explored a virtual environment and later drew its sketch-map. Our results showed participants spent more time and perceived less motion-sickness and task effort using desktop than HMD VR. With reduced interaction fidelity, the high visual fidelity of HMD as compared to desktop resulted in similar or poorer performance on different spatial learning tasks after accounting for motion-sickness and workload effort. Participants were better in recalling spatial components related to junction and cyclic order of the navigated virtual space in desktop vs. HMD VR, and performed equally well on components related to street segments and object associations. We explain these results in terms of deficient idiothetic information in non-ambulatory HMD and lesser sensory conflicts in desktop mode. Overall, our results highlight the differential effect of visual vs. interaction fidelity on human performance based on using immersive technologies, how such an effect depends on the nature of cognitive and functional behavior users employ, and the higher usability of traditional desktop VR. These results are relevant for developing customized and sustainable virtual reality based human-computer interactions.

## Introduction

Making sense of space is one of the fundamental exercises we perform every day, such as wayfinding, reading and interpreting visual maps, planning and utilizing mental maps, reaching out for or grasping and holding objects, etc. Processing of spatial information during acquisition of spatial knowledge, and especially during navigational tasks involving wayfinding, depends on a variety of factors, such as the scale of the environment (Waller and Nadel, 2013), spatial cues (Wolbers and Hegarty, 2010; Waller and Nadel, 2013), self-motion or idiothetic cues (Chrastil and Warren, 2013, 2014, 2015; Murcia-López and Steed, 2016), cognitive abilities (Chrastil and Warren, 2012, 2013, 2015; Waller and Nadel, 2013), mental representation (Gillner and Mallot, 1998; Klatzky, 1998; Wolbers and Hegarty, 2010; Waller and Nadel, 2013), self-sense of direction (Davies et al., 2017), and previous experiences (Dabbs et al., 1998; Feng et al., 2007; Green and Bavelier, 2007; Boot et al., 2008; Spence and Feng, 2010; Adamo et al., 2012). Recent proliferation of virtual reality (VR) technology has suggested new factors or re-evaluated the importance of already known factors that might influence spatial learning in a virtual environment (VE), such as display modalities (e.g., desktop or DT, Head Mounted Display or HMD, CAVE), optical distortion, field of view (FOV), visual and interaction realism, and visual and motion latency (Loomis and Knapp, 2003; Knapp and Loomis, 2004; Interrante et al., 2006; Toet et al., 2008; Boonsuk et al., 2012; van der Ham et al., 2015; Wilson and Soranzo, 2015; Jerald, 2016; Sahu et al., 2017; Roettl and Terlutter, 2018). In the present study, we investigated how two different forms of display, desktop (DT) and head mounted display (HMD), affect spatial knowledge learned during a navigational task in a VR setting.

### Spatial Learning in VR Settings: Desktop vs. HMD

VR has become one of the fastest growing fields for research, development, and entertainment (Ardouin et al., 2012; Boonsuk et al., 2012; Chrastil and Warren, 2013, 2014, 2015; Wilson and Soranzo, 2015; Murcia-López and Steed, 2016; Roettl and Terlutter, 2018). The chief advantage that VR provides researchers and developers is to be able to create a setting with a high degree of control and treatment manipulation. It provides new research avenues with umpteen possibilities to create stimuli that are more ecologically valid and to collect various types of response executed in different modalities (Wilson and Soranzo, 2015). This would help us to understand human cognition and behavior, and to also train them, for example, for military combat and reconnaissance, medical surgical operations, driving scenarios, etc. However, despite significant advancement in VR technologies to simulate the real environment, a substantial gap still exists in mapping realism onto the virtual world. Research shows that not only this gap but also even a high degree of mapping between the real and virtual world, for example, in terms of immersiveness, may not be very effective or could be even detrimental for human learning and performance. A more ecologically valid setting may not always produce more ecologically valid responses.

Advancement in display technologies has made accessible to the general public the once expensive and rare devices that can effectively and closely simulate the real environment. One of the most typical and fundamental properties of VR systems is presentation of stimuli in three dimensions. This is generally done on a flat computer monitor (DT), HMD, or room-like cubical (CAVE). Of these, DT and HMD, which are increasingly becoming more affordable consumer products, have also been commonly studied by researchers to examine their comparative effect on human cognitive and functional behaviors. Spatial learning has been commonly investigated with VR technology. Both immersive (e.g., HMD) and non-immersive (e.g., DT) VR devices have been used for this purpose, however, it is not clear if one has an advantage over the other (Stevens et al., 2015). We discuss below two factors, immersion and VR induced symptoms and effects (or VRISE; Sharples et al., 2008), that are relevant to the efficacy of immersive devices for research purposes, as they may differentially affect the usability of DT and HMD as research set-ups for studying spatial learning.

### Presence, Immersion, and Fidelity

One of the most fundamental goals of VR technology for both developers and researchers is to have the consumer of VR or participants in a research setting have optimal experience of the real world through virtual means. This experience generated through VR technology is a subjective psychological factor, generally termed as “sense of presence” or simply “presence”—“Presence is a state of consciousness, the (psychological) sense of being in the virtual environment” (Slater and Wilbur, 1997, p. 4) and may not correspond in a one-to-one manner with the changes in the virtual environment. This subjective psychological factor, however, is sometimes confounded with an objective technological factor (Slater, 2003) called “immersion.”

Immersion is one of the most important technology and design factors for VR. Slater and Wilbur (1997) provided a clear and comprehensive definition of immersion to be “a description of a technology, and describes the extent to which the computer displays are capable of delivering an inclusive, extensive, surrounding, and vivid illusion of reality to the senses of a human participant” (p. 3). This comprehensive definition makes it very clear that immersion “stand[s] simply for what the technology delivers from an objective point of view” (Slater, 2003, p. 1.) Any psychological factor, whether what the VR users experience, or any cognitive process they use, such as navigational planning or strategy, is typically not defined as a part of immersion. Whereas, the VR users' experience is related to presence, navigational planning is related to a different factor called “involvement.”

An earliest and intuitive goal to improve VR technology was to achieve maximum realism through virtual experience. Fidelity is the term used to describe the degree of resemblance between the virtual and real objects and actions (Slater, 2003; Jerald, 2016; Murcia-López and Steed, 2016). It is directly related with immersion and presence—in that, as a VR system provides better multimodal displays and tracking, its fidelity increases, which results in a more immersive VE, which thereby evokes a stronger sense of presence. Fidelity is made up of display fidelity and interaction fidelity, where display fidelity refers to the degree to which the virtual display features (e.g., a DT monitor or HMD) conform to the real-world features. Interaction fidelity refers to the degree to which the sensory-motor feedbacks conform to the real-world interaction (e.g., how optic flow changes while walking and driving in a VE). Higher fidelity makes a VE more immersive, with the goal that it will elicit more realistic psychological responses, or evoke a better sense of presence. However, research shows it is not clear how helpful a higher than lower level of immersion is for performance on various cognitive and functional tasks (Slater et al., 1994; Wilson and Soranzo, 2015).

Immersion results from an interplay of multiple factors, which includes use of multiple modalities, inclusiveness by shutting out the physical reality of users, vividness of resolution and fidelity, the extent of body or head tracking and matching it with the changes in the display, intractability, and story plot (Slater et al., 1994; Slater and Wilbur, 1997; Witmer and Singer, 1998; Jerald, 2016). To better understand how immersion affects human behavior, for example, during spatial learning, the individual effects of the related constituent factors of immersion and their interrelations must be understood. Studies looking at the effect of different display modes, for example, generally manipulate display fidelity, sometimes alongwith varying combination of interaction fidelity (McMahan et al., 2012; van der Ham et al., 2015; Murcia-López and Steed, 2016; Roettl and Terlutter, 2018). These studies allowed participants to exercise different kinds of control to navigate in a VE. For example, some studies with HMD allowed ambulatory navigation while in others, participants had to sit on a chair and control the optic flow with combinations of keyboard, joystick, or hand-held sensing and steering controllers. Body movements play an important role in navigation, and change the interaction fidelity of the VR system, which could affect the sense of presence and performance (Chrastil and Warren, 2012, 2013; Berger and Wolf, 2018). Any systematically varying but non-manipulated restriction on body movements across experimental conditions can produce confounding. Therefore, for a clearer understanding of the effect of immersion on spatial learning during navigating a virtual space, we have to ascertain the individual contribution of display and interaction fidelity.

HMD compared to DT VR is more immersive because of the HMD system's ability to disconnect with the physical local environment by blocking eye-contact with the real-world objects, and to provide self-motion feedbacks, such as those involving head and body movements cues. These feedbacks foster utilizing real-world strategies while exploring the VE and therefore should lead to better sense of presence and performance with HMD than less immersive VR systems (Slater, 2003; McMahan et al., 2012; Murcia-López and Steed, 2016). We investigated how two different display modes, DT and HMD, affect spatial learning during navigation in a VR setting. These display modes differ at the level of visual realism they generate because of the different degree of ego-centric visual feedbacks they provide during navigation.

### VR Induced Symptoms and Effects

Virtual environment offers many advantages for studying spatial learning but has its own unique challenges arising from higher degree of immersiveness, such as various kind of sickness and increased workload, which must be taken into account if we want to use this fast growing technology for larger applicability, usability, precision, and control. One of the biggest challenges for immersive technology is to control motion sickness (Sharples et al., 2008; Wilson and Soranzo, 2015; Jerald, 2016; Lu, 2016; Berger and Wolf, 2018; Roettl and Terlutter, 2018). VR sickness, a visually induced motion sickness, results from immersion in a computer-generated virtual world (Sharples et al., 2008; Wilson and Soranzo, 2015; Jerald, 2016; Lu, 2016; Berger and Wolf, 2018). Studies evaluating VR sickness show that it is a multifactorial problem that could be caused by any of the VR technical features, such as those related with FOV, optical distortion, flicker, refresh rate, resolution, latency and poor tracking of visual and interaction feedback, slow update rate, etc. (Sharples et al., 2008; Toet et al., 2008; Wilson and Soranzo, 2015; Jerald, 2016). A higher realism graphics causes more visual flow and stronger sensory conflict that could cause VR sickness (Jerald, 2016).

Sharples et al. (2008) coined a term, VRISE, for VR induced symptoms and effects, as a cumulative description of various symptoms and side-effects resulting from the immersiveness of VR systems. These may include feeling of nausea, disorientation, and oculomotor sickness such as eye strain and headache. Sharples et al. (2008) show a higher rate of VRISE in HMD compared to DT, projector screen and reality theater. Other studies comparing DT and HMD VR have reported increased demand of cognitive and physical workload (Lu, 2016; Roettl and Terlutter, 2018) while using HMD VR which could affect performance. Both VRISE and increased workload could make the VR technology inefficient for human use and may produce disruptive “uncanny” experiences (Mori, 1970). Studies that compare the usability of different degree of immersiveness of VR devices must examine the comprehensive experience that users have in VR settings. It must be ascertained what role VRISE and workload play in findings that show or fail to show better performance in a more than less immersive VR system.

Murcia-López and Steed (2016) found better spatial learning performance in HMD than DT system. They did not find any difference across the two conditions on self-reported measures of subjective experiences as a result of virtual navigation. These measures included survey items on confidence, difficulty, and movement. They found that HMD in high fidelity condition is better for spatial learning than DT. These findings indicate that if an immersive technology does not result in VRISE and reduce workload, they may result in optimal experience and improve performance. However, it is still not clear if most studies are successful in controlling the effect of such disruptive factors, and why in Murcia-López and Steed's study, participants did not experience the commonly reported VR related discomfort or sickness. One reason could be that Murcia-López and Steed used only two survey items (examination, and movement) that indirectly measured participants' subjective experience of VR induced discomfort. A more comprehensive measure that differentially assess motion sickness and task related effort could throw more light on how these variables affect performance in a VR setting. In the present study, we used a detailed assessment of both motion sickness and workload during a virtual navigation task.

### Related Research

Many empirical studies have investigated the comparative effect of DT vs. HMD VR on performance enhancement on different tasks related to cognitive and functional behaviors. Results have been mixed as a substantial proportion of studies show the intuitively advantageous appeal of high immersion of HMD VR actually results in worse performance. For example, Santos et al. (2009) used a randomized within-subject design to compare navigational performance in HMD vs. DT VEs. They found that participants made same number of collisions in the two VR conditions but caught more objects, were faster and traveled larger distances in the DT than HMD condition. To navigate in the VE, participants used their head movements and hand-held mouse in the HMD condition, and mouse and keyboard in the DT condition.

Roettl and Terlutter (2018) had participants play “jump‘n’run” video games in three conditions: 2D, stereoscopic 3D, and HMD VR. They found best recall and recognition of placed brands in the 2D condition, and worst in the HMD VR condition. HMD VR also resulted in highest cognitive load (lowest in 3D), dizziness (lowest in 2D), and motion-sickness while playing (lowest in 2D). Other studies also show weaker support for HMD based training when compared to standard computer monitor (Manrique, 1998), or the real world (Hamblin, 2005). McMahan et al. (2012) showed how display fidelity and interaction fidelity interact to affect video game performance. Higher accuracy was observed in both low display fidelity as well as low interaction fidelity conditions than their higher counterpart conditions. To sum up, it could be concluded that currently there is no clear-cut support for beneficial effects of higher immersive VR technology over less immersive ones, and a good number of studies have showed detrimental effects of the immersive technology (for overall review see Stevens et al., 2015).

Some studies have also reported an advantage of using HMD over DT VE, for example, in performance on specific cognitive ability tasks (Parmar et al., 2016), and spatial judgement task (Murcia-López and Steed, 2016). These studies varied the interaction fidelities by using controls, such as keyboard or motion-sensing game controllers, or by allowing locomotion. However, Parmar et al. (2016) also found that participants' performance on different cognitive ability tasks showed equal improvement in DT and HMD conditions, and performance on psychomotor tasks was better in DT vs. HMD condition. The participants in this study were trained using 3D simulation to learn about electrical circuitry. Participants did show better learning in HMD than DT for higher level concepts related to task evaluation but also took more time and traversed more distance in HMD vs. DT condition. In both the study conditions, they had to sit on a chair and interacted with the VE using motion and orientation sensing game controls held in both hands.

Murcia-López and Steed (2016) found that by navigating in a high detail VE with high visual fidelity, participants acquired better spatial learning in high immersion (i.e., HMD) than in low immersion (i.e., DT) condition. For conditions relevant to the present study, their participants learned positions of three identical objects by navigating virtual space through either DT or HMD. Spatial learning was assessed by the accuracy of placing the physical replicas of these objects in a real room with same configuration of the virtual room. However, the high and low immersive conditions in their study differed not just on visual fidelity but also interaction fidelity—participants explored the VE by engaging in natural physical walking in the HMD condition but in the DT condition they were allowed to use only keyboard and mouse. It is not clear whether results from Murcia-López and Steed (2016) are due to the difference in the kind of displays used in the two immersive VEs (low vs. high visual fidelity) or due to the difference in the level of possible active navigation in these VEs.

One likely reason why natural walking was allowed in the high immersion condition in Murcia-López and Steed (2016) could be because of the way immersion was conceptualized by the authors to involve both the display types and also the navigational techniques: “We also consider the navigation technique associated with each learning system as an inherent and crucial element of level of immersion” (Murcia-López and Steed, 2016, p. 3). However, navigational techniques are psychological construct used actively by participants. Murcia-López and Steed's definition of immersion seems less typical because immersion has been generally defined as a technology-driven objective factor without an inclusion of active (psychological) navigation techniques (Slater and Wilbur, 1997; Witmer and Singer, 1998; Jerald, 2016). Notwithstanding that the more typical definition of immersion is related to the VR technology and not the cognitive processes of the users, even if the conception of immersion were to be modified to include the active navigation technique besides display features, it would be imperative for a functional understanding of immersion to tease apart the individual effects of various elements that constitute immersion, such as visual vs. interactional fidelity.

As discussed above, previous research has shown that ambulatory conditions (in HMD) is advantageous than the locomotion-restricted condition (of DT) because locomotion provides richer and more useful information coming from a navigator's motor, proprioceptive, and vestibular system (Chrastil and Warren, 2012). In the present study, we aimed to understand if results in the HMD condition in Murcia-López and Steed (2016) study could have been caused by allowing walking, as it would provide ambulatory information. To investigate that, we did not allow walking in the HMD condition in the present study, and allowed the same computer peripheral controls (i.e., Xbox 360) for virtual navigation in both the HMD as well as DT conditions. However, we did allow head movement for viewing purpose in the HMD condition for participants to not feel—constricted. Specifically, in case of HMD, we allowed participants to use head rotation for visual scanning in the right/left and up/down directions but use only Xbox 360 controller for moving forward/backward and rotating left/right during locomotion in the virtual space. This manipulation allowed us a tighter comparison of HMD to DT to infer how immersion, with visual fidelity but with low interaction fidelity, would affect spatial learning during virtual navigation.

### Present Study

In the present study, we evaluated the effect of non-ambulatory HMD vs. DT on spatial learning during a virtual navigation task. We used a 1,500 × 2,000 m outdoor virtual environment that was developed in the videogame developing platform Unity 3D, using first person shooter's perspective. Our goal was to understand how would visual fidelity in the two display conditions affect spatial learning. To disentangle the effect of visual and interaction fidelity in HMD and DT VR, we used similar virtual locomotive interface, that is, Xbox 360 controller, for both the display modes. Since high visual and interaction realism has been associated with detrimental psychophysiological effect, we aimed to evaluate the role of disruptive features of VR, such as VRISE and workload, in the difference in spatial learning. The two display modes were compared on spatial learning, time taken to compete the virtual exploration, motion sickness, workload, sense of presence, and spatial orientation ability. It was hypothesized that if idiothetic cues play an important role in using HMD VR, then restricting ambulatory interaction will impede the virtual navigation in HMD and therefore virtual spatial learning.

## Methods

### Participants

Forty undergraduate and graduate students (27 males: 14 in DT and 13 in HMD; 12 females: 6 in DT and 6 in HMD; and 1 other category in HMD; mean age = 22.6 years, SD = 2.41 years) from International Institute of Information Technology–Hyderabad (IIIT-H), India participated in the study. They reported having normal or corrected-to-normal vision, with minimal or no interaction with HMD devices. The demographics of participants were decided by who voluntarily decided to participate in the experiment. Written informed consent was obtained from all participants and they received monetary compensation for their participation. The study protocol was approved by IIIT-H's Institutional Review Board (IRB) Committee.

### Design

We manipulated mode of display of the VE to create two treatment conditions: DT and walking-restricted or non-ambulatory HMD (henceforth, HMD). Forty participants were randomly assigned across the two conditions, and were administered the same instructions, practice and experimental tasks.

### Virtual Reality System

The experiment was conducted in a Virtual Human Interaction Lab at IIIT-H. We used Alienware 15' gaming laptop with 1,920 × 1,080 resolution, NVIDIA GeForce GTX 1060 graphics card, and 60 Hz refresh rate to render the virtual environment for HMD. We used Oculus rift Unity 3D standard package for building the layout. The oculus rift CV1 projected 1,080 × 1,200 pixel resolution per eye, and 2,160 × 1,200 for both eyes, whereas the DT consisted of 1,920 × 1,080 resolution. We used HP Z238 Microtower workstation, with NVIDIA Quadro K420 graphic controller, and Dell 24' monitor with 1,920 × 1,080 resolution to render the DT virtual environment.

For the HMD condition, all participants viewed 110° stereoscopic FOV that consisted of 1,080 × 1,200 pixel resolution per eye, with 90 Hz refresh rate and 360° positional tracking. The oculus rift CV1 optical 360° tracking sensor enabled tracking of head position and orientation in real space and translated the same to virtual space, with <5 ms latency and with 1,000 Hz rotational and 60 Hz positional update rate. Participants were instructed to use head rotation to look right/left and up/down to scan the virtual environment but use Xbox 360 controller for moving forward/ backward and rotating left/right for navigating the virtual environment.

In the current study, we varied the visual fidelity of the two display modes and investigated its impact on spatial learning, motion sickness, mental workload, and sense of presence. The virtual locomotion interface was kept similar across both the display modes. We used Xbox left-stick for translational movement and Xbox right-stick for the rotational movement. Virtual navigation in HMD condition enabled stereo-perception, and egocentric spatial reference because of the visual feedback in response to head rotation. Comparatively, navigation in DT VR would use more monocular cues and less egocentric spatial reference.

All participants in DT condition viewed 2D display that consisted of 1,920 × 1,080 resolution, 60 Hz refresh rate to project the 110° FOV of the virtual environment. We used three virtual cameras in Unity 3D with stereo separation at 0.022° and stereo convergence at 10° targeting both eyes, and used NVIDIA Quadro K420 graphic controller to render the virtual environment. In DT condition, the average speed was 10.73 m/sec, SD = 1.54 m/sec, and in HMD condition the average speed was 5.37 m/sec, SD = 0.97 m/sec. Before starting the main experiment, an informal pilot study was conducted to compare the comfort in speed of movement in both the display modes.

### Virtual Environment

**Figure 1:**
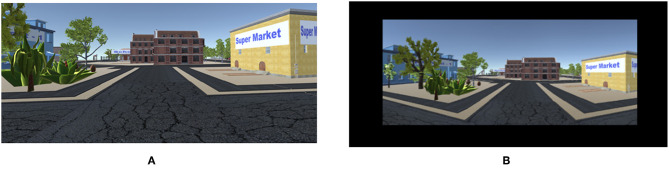
First person shooter's perspective from **(A)** HMD, and **(B)** DT.

The virtual environment was developed in the videogame developing platform Unity3D at 60 frames per second (fps) on Windows 7OS, using first person shooter's perspective (Figure 1). In both the conditions the virtual cameras projected 110° FOV of the virtual environment on the corresponding display modes. In HMD condition the virtual camera FOV matched the HMD FOV. However, in DT condition, the virtual camera was not aligned with the viewers' 110° FOV. In DT condition, the 110° virtual FOV was subtended at a diagonal angle of 19° from 80 cm seating arrangement. The distance between the virtual agent and the viewed object at a particular virtual spatial position were the same (Figure 1). The experiment was conducted in a dimly lit, sound-proof room.

**Figure 2:**
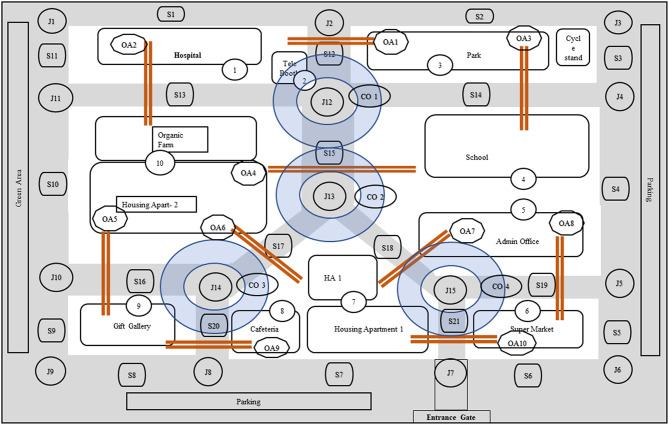
Virtual environment layout.

The 1,500 × 2,000 m virtual environment contained 21 routes, 15 junction points, and 10 unique objects of interest, such as a park and different building structures with unique but familiar names, and four landmarks, such as the two parking areas and enterance gate (Figure 2). A street segment was defined as path or route between two junctions. For example, in Figure 2, street segment S1 is between junctions J1 and J2, and street segment S2 is between junctions J2 and J3. A junction was defined as a node where two or more street segments meet, such as junction J1 in Figure 2, where street S11 and S1 meet.

The VE also contained 7 regional blocks, each consisting of either one or two building structures within the block area. Fifteen junctions comprised four “L” junctions, seven “T” junctions, one “+” or cross junction, one “Y” junction, and two “semi Y” juctions. The + junction, Y junction, and two semi-Y odd junctions were considered as special junctions for the recall task because of their spatial association with streets and various unique objects in the VE, such as a park and different building structures with unique but familiar names. The spatial configuration of such junctions in clockwise direction was named as “cyclic order” (Wang and Schwering, 2015). The virtual layout contained 4 cyclic orders (Figure 2).

### Procedure

**Figure 3:**
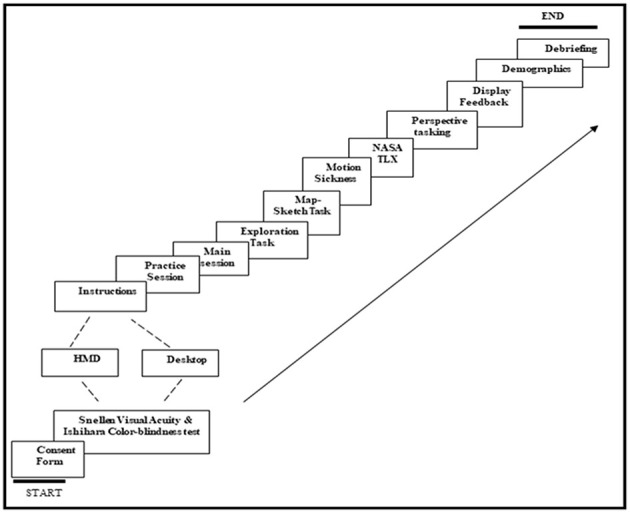
Experiment schematic flow.

Figure 3 depicts the procedure for an experimental session. Each study session with a participant started with administering vision related screening tests: Snellen visual acuity and Ishihara colorblindness tests. Participants were then randomly assigned to either the HMD or DT treatment condition. They first went through a practice session to familiarize themselves with the virtual interface and the corresponding key controls. The VE layout was simpler in the practice than experimental session. In the practice session, the layout contained 4 unique objects, 9 junctions, 12 street segments, and only one cyclic order at a cross junction. The greater complexity of the VE in the experimental session is described above in the section Virtual Reality Induced Effects, Presence and Perspective Taking. The practice session was followed by the experimental session, which consisted of three tasks administered in the following fixed order: an exploration task, a sketch-map task, and a set of five surveys.

Participants received same instructions in both the experimental display conditions. They were instructed to navigate the VE to learn its layout and the spatial locations of various objects. They were informed that after completing the exploration they will be asked to draw a map of the layout from their memory of the exploration. Participants started the exploration from an “Entrance Gate” (see Figure 2). They were instructed to return to this entrance gate after the completion of the exploration. They were allowed maximum 15 min for the exploration however they could finish the exploration before this time if they felt they had sufficiently explored the VE. In both the display conditions, participants navigated the VE using the X-box 360 controller, which had separate keys for translational and rotational movements. However, the scanning of the VE was different in both the display modes. In HMD condition, participants were allowed to use the head rotation: up/down and right/left to scan the virtual environment, whereas in DT condition participants scanned the VE using Xbox 360 controller to rotate and change the field of view in the given VE while sitting at a fixed location.

**Figure 4:**
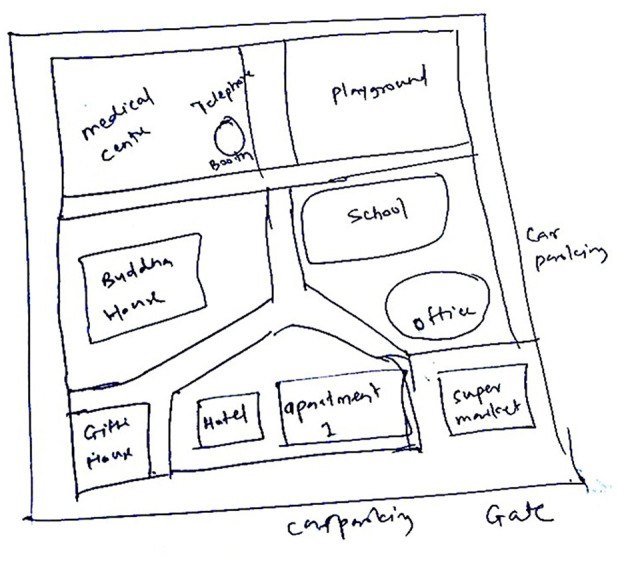
An example of sketch-map drawn by a participant after virtual exploration.

After the exploration task, participants completed a sketch-map task based on their memory of the navigated VE. They reconstructed the layout of the VE and the locations of the virtual objects by sketching a map on a given A4 size white paper (see Figure 4 for an example of a sketch-map drawn by a participant). The performance on the sketch-map task was evaluated by comparing its spatial components with those of the explored VE.

We administered a set of five surveys as the last task of the experimental session. These surveys measured: subjective task workload with NASA-Task Load Index (TLX) assessment tool (NASA, 1986; Hart and Staveland, 1988), sense of presence with the presence questionnaire by Witmer and Singer (1998), motion sickness with Kennedy Questionnaire (Kennedy et al., 1993; Sharples et al., 2008), and spatial ability with the Perspective Taking/Spatial Orientation Task (PTSOT) by Kozhevnikov and Hegarty (2001). The last survey gathered participants' demographic details.

### Measure of Performance

The present study compared two display modes, DT vs. HMD, on a number of dependent variables: spatial memory of the explored VE, total time taken to explore the VE, motion sickness, subjective workload, sense of presence, and perspective taking or spatial orientation ability.

#### Sketch-Map Scoring

Sketch-maps provide valuable details about invariant spatial information learned during exploring an environment regardless of the general cognitive distortions and schematization (Wang and Schwering, 2015). A freehand drawn sketch map is a better measure of spatial learning than absolute measures, such as Euclidian distances and angles, because drawing a sketch map involves using a navigator's egocentric, local metric information of the navigated space, which have been shown to be more compatible with human spatial knowledge than globally consistent absolute spatial knowledge (Gillner and Mallot, 1998; Warren et al., 2017).

In the present study, we measured participants' spatial knowledge learned during the virtual exploration by scoring their sketch map for recall of spatial relationship between three sets of spatial items, which includes routes, junctions and unique objects of interest. For analyzing the sketch-maps, we used qualitative techniques to measure topology, orientation and order of spatial items by closely following the scoring procedure used by Wang and Schwering (2015) and Billinghurst and Weghorst (1995). Readers are referred to these two studies for the underlying assumptions and further details of the scoring process. Accuracy of learned spatial knowledge was computed by scoring the degree of match between participants' sketch-map and the explored VE's layout for the following four types of spatial relationships:

1. *Topology of street segments* (SS)—This referred to the connectivity between street segments in a given street network. For example, S_1_ and S_2_ (Figure 2) were considered connected if and only if they shared at least one junction, otherwise they were considered as not connected. There were total 21 street segments. The maximum raw accuracy score for correctly recalling SS was 21.
2. *Topology of the junctions* (JXN)—This referred to the connectivity between junctions in a given street network. For example, J1 and J2 were connected if there was an intermittent street between them. There were total 15 junctions, and maximum raw accuracy score for JXN was 15.
3. *Topology of object associations* (OA)—This referred to the spatial relation between two proximal objects, which could be either opposite to each other (e.g., O1 and O3) or parallel (e.g., O1 and O10). The object association between O1: hospital and O3: park was called as OA1, and object association between O1: hospital and O10: housing apartment with organic farm was called as OA2. Such spatial relationships were counted only when they connected a street segment in between two landmarks. There were 10 such associations (Figure 2). The maximum raw accuracy score for OA was 10.
4. *Cyclic order of street segments and objects around a junction* (CO)—This refered to the local network between street segments and objects with respect to an adjacent junction. There were four cyclic orders between street segments and objects at a given junction. We designed three kinds of junctions: a junction with four paths intersecting at 90°; a Y junction with three paths intersecting at 120°; and two skewed Y junctions with three paths intersecting at 150, 120, and 90° (see Figure 3). There were total four cyclic orders, the first CO was at “+” or cross junction, second CO was at Y junction, and third and fourth COs were at the skewed Y junctions (Figure 2). The junction intersections were varied to have a complex layout with a diverse set of orientations across different angular intersections. The maximum raw accuracy score for CO was 4.

We calculated accuracy scores for the four separate spatial relations (i.e., SS, JXN, OA, and CO). The inter-rater reliability for blind raters of the scores for the correct recall of the spatial relations was 0.91. We did the separate evaluations of the four spatial components because each spatial relation has its unique roles in spatial memory—whereas the topology of the street segments and junctions reveals the geometric aspect of a layout, the topology of object association and cyclic order reveals the feature-based aspect of a given environment (Wiener et al., 2009; Wolbers and Hegarty, 2010; Waller and Nadel, 2013; Murcia-López and Steed, 2016). This information allows evaluation of the advantage that one specific spatial component might have over the other in building spatial memory when navigation different VEs, such as HMD vs. DT.

#### VRISE Scoring

Simulator sickness values were calculated using the Kennedy simulator sickness questionnaire (Kennedy et al., 1993; Sharples et al., 2008). This questionnaire has 15 items. Each item is constructed using the following four-point Likert scale, where 1 = None, 2 = Slight, 3 = Moderate, and 4 = Severe. The dimensions of sickness included oculomotor, nausea, and disorientation. Each participant's overall simulator sickness was calculated by averaging the ratings on all the items. Similar calculation was performed for NASA TLX and Sense of Presence Surveys as well. VR interaction workload values were calculated using the NASA TLX assessment tool (NASA, 1986). This questionnaire has six items. Each item is constructed using a seven-point Likert scale, where 1 = very low, and 7 = very high. The dimensions of workload included mental demand, physical demand, temporal demand, performance, effort, and frustration. An average of the responses on all the items provided a participants NASA TLX score. Sense of presence values were calculated using the Witmer and Singer (1998) Questionnaires. These questionnaires have 15 items on sense of within virtual reality environment and 6 six items on sense of outside virtual reality environment. Each item is constructed using a five-point Likert scale, where 1 = strongly agree, and 5 = strongly disagree. Each participant's overall sense of within and outside virtual reality environment sense of presence was calculated by averaging the ratings on all the items.

## Results

We analyzed the data to first examine if the two display conditions resulted in different degree of correct recall of the four spatial components of the navigated VE. Since the four components represented different spatial information that were not directly comparable and because the maximum number of items that could be correctly recalled was different in each condition (SS = 21, JXN = 15, OA = 10, CO = 4), we studied the effect of display condition separately on these spatial components by performing one-way ANCOVA for recall of each spatial component, with motion sickness and workload as covariates in these analyses. We then performed independent-sample *t*-tests to look at how the two display conditions differed with respect to the total time taken to complete the task, motion sickness, workload, sense of presence, and perspective taking task performance. Before starting the analysis, we looked for any outliers using the lower bound cut-off criterion of M – 2SD, and upper bound cut-off criterion of M + 2SD. We found eight outliers for six dependent variables across the display condition groups. We also found violations of the assumption of normality and homoscedasticity for some of the conditions. To be consistent with the analysis across the comparison groups and to deal with the violation of statistical assumptions for ANCOVA and *t*-test, we decided to use 20% trimmed means for all the statistical analysis we performed. Such an analysis helps in computing robust statistics that provide improved power and improved control over the probability of Type I error for data with outliers and violations of assumption of general linear model statistics (Wilcox and Rousselet, 2018).

### Topological Spatial Knowledge

**Figure 5:**
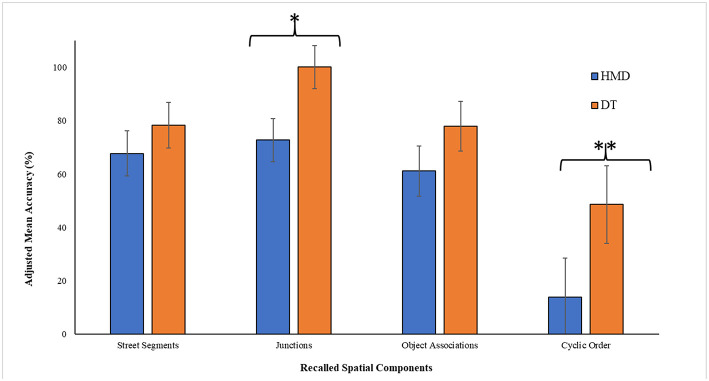
Adjusted mean accuracy on the map-sketch task for recalling four different spatial components of the navigated virtual environments in head mounted display (HMD) vs. desktop (DT) conditions. The accuracy scores are adjusted for two covariates: motional sickness and workload. Error bars represent 95% CIs. ^*^ denotes *p* < 0.05; ^**^ denotes *p* < 0.01.

Learned topological spatial relationships were analyzed by conducting a one-way ANCOVA to study how display condition (DT vs. HMD) affected the accuracy of recalling four different components of the navigated VE (i.e., SS, JXN, OA, and CO) after controlling for VR induced sickness (or VRISE) and workload in performing the virtual navigation task (as measured by the NASA TLX assessment tool). All the raw scores were converted into percentage scores. We used 20% trimmed means to compute robust statistics for each of the spatial learning component. Figure 5 shows mean accuracy for the recall of the four spatial components across the two display conditions.

*Street Segments (SS)* The adjusted mean for SS was higher in for DT (adjusted *M*_DT_ = 78.315, *SE*_DT_ = 4.075) than HMD (adjusted *M*_HMD_ = 67.750, *SE*_HMD_ = 4.075) but this difference was not significant *F*(1, 20) = 2.532, *p* = 0.127, η_*p*_^2^ = 0.112. None of the covariates had a significant effect: motion sickness, *F*(1, 20) = 0.037, *p* = 0.850, η_*p*_^2^ = 0.002; Workload: *F*(1, 20) = 0.259, *p* = 0.616, η_*p*_^2^ = 0.013.

*Junctions (JXN)* We found a significant effect of display condition on the recall of Junctions, with better recall in DT (adjusted *M*_DT_ = 84.422, *SE*_DT_ = 3.458) than HMD (adjusted *M*_HMD_ = 71.920, *SE*_HMD_ = 3.458) condition, *F*(1, 20) = 4.751, *p* = 0.041, η_*p*_^2^ = 0.192. None of the covariates had a significant effect: motion sickness, *F*(1, 20) = 0.791, *p* = 0.384, η_*p*_^2^ = 0.038; Workload: *F*(1, 20) = 0.001, *p* = 0.982, η_*p*_^2^ < 0.001.

*Object Associations (OA)* We found a significant effect of display condition on recalling of Object Associations, with better recall in DT (adjusted *M*_DT_ = 77.902, *SE*_DT_ = 4.489) than HMD (adjusted *M*_HMD_ = 61.125, *SE*_HMD_ = 4.489) condition, *F*(1, 20) = 5.451, *p* = 0.030, η_*p*_^2^ = 0.214. None of the covariates had a significant effect: motion sickness, *F*(1, 20) = 0.038, *p* = 0.847, η_*p*_^2^ = 0.002; Workload: *F*(1, 20) = 1.703, *p* = 0.207, η_*p*_^2^ < 0.078. However, we found that the assumption of homogeneity of error variance, as revealed by the Levene's Test, was violated, *F*(1, 22) = 6.452, *p* = 0.019. We transformed the data using the inverse function which resulted in having homogeneity of error variance assumption accepted, *F*(1, 22) = 3.416; *p* = 0.078, however, with the transformed data, display condition did not have a significant effect on the recall of Object Association, *p* = 0.067.

*Cyclic Order of Street Segments and Objects Around a Junction (CO)* Display condition had a significant effect on the recall of Cyclic Order, with better recall in DT (adjusted *M*_DT_ = 48.629, *SE*_DT_ = 6.983) than HMD (adjusted *M*_HMD_ = 13.871, *SE*_HMD_ = 6.983) condition, *F*(1, 20) = 9.559, *p* = 0.006, η_*p*_^2^ = 0.323. None of the covariates had a significant effect: motion sickness, *F*(1, 20) = 0.426, *p* = 0.521, η_*p*_^2^ = 0.021; Workload: *F*(1, 20) = 0.241, *p* = 0.629, η_*p*_^2^ < 0.012. However, we found that the assumption of homogeneity of error variance, as revealed by the Levene's Test, was not met, *F*(1, 22) = 15.075, *p* = 0.001. We transformed the data using the log_10_ function, which resulted in a non-significant Levene's test, *F*(1, 22) = 3.068, *p* = 0.094. Similar to the results with non-transformed data, also for the transformed data, we found that display condition had a significant effect on recall of cyclic order, *F*(1, 20) = 11.624, *p* = 0.003, η_*p*_^2^ = 0.368. None of the covariates had a significant effect: motion sickness, *F*(1, 20) = 0.1.261, *p* = 0.275, η_*p*_^2^ = 0.059; Workload: *F*(1, 20) = 0.038, *p* = 0.847, η_*p*_^2^ = 0.002.

### Total Time Taken

**Figure 6:**
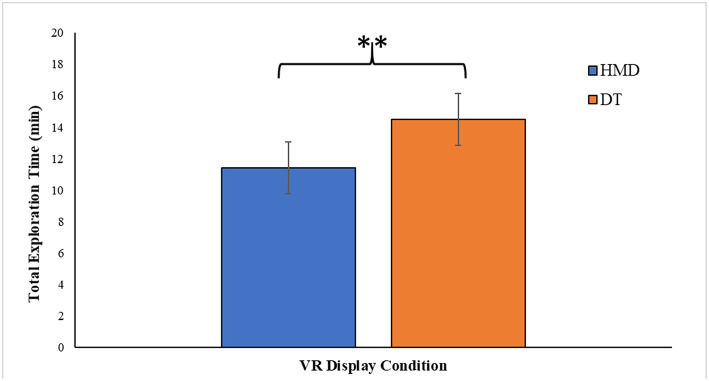
Total exploration time in minutes for the two VR display conditions: head mounted display (HMD) vs. desktop (DT). Error bars represent 95% CIs. ^**^ denotes *p* < 0.01.

Total time taken was calculated as either the number of minutes participants took to complete the virtual exploration by returning to the starting point, or as 15 min, when the participants used up this maximum time allowed for exploration. We analyze the data with 20% trimmed means. We observed a significant effect of display mode on total exploration time, *t*(12.886) = 4.011, *p* = 0.002, Cohen's d **=** 1.71, after adjusting for degrees of freedom, as Levene's test for equality of variances was significant *t*(22) = 21.682, *p* < 0.001. Participants explored the VE longer when it was rendered by a DT display (*M* = 14.49, *SE* = 0.215) than HMD (*M* = 11.43, *SE* = 0.732) (Figure 6).

### Virtual Reality Induced Effects, Presence, and Perspective Taking

**Figure 7:**
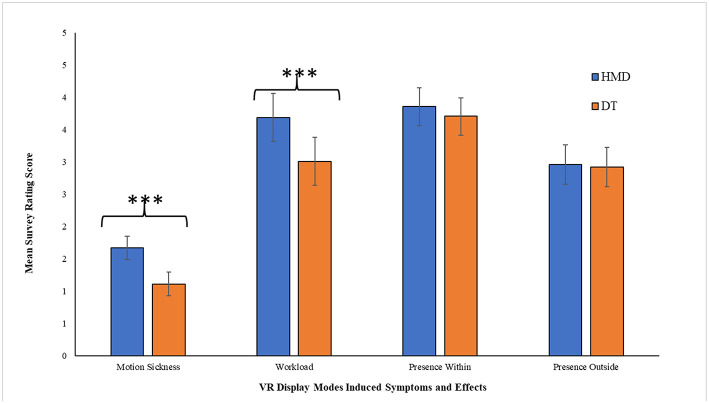
Mean of raw rating scores on survey measures of different symptoms and effects induced by the two VR display conditions: head mounted display (HMD) vs. desktop (DT). Error bars represent 95% CIs. ^***^ denotes *p* < 0.001.

We analyze the data for each dependent variable with 20% trimmed means. Display mode showed a significant effect on virtual reality based motion sickness, *t*(13.369) = 6.304, *p* < 0.001, Cohen's d = 2.71 (Figure 7); Levene's test: *t*(22) = 6.304, *p* = 0.001. HMD condition induced higher motion sickness (*M* = 1.67, *SE* = 0.084) than DT (*M* = 1.11, *SE* = *0*.028). The task load measure, assessed using NASA-TLX measure, showed that participants subjectively assessed themselves as putting in more effort for virtual exploration in the HMD condition (*M* = 3.69, *SE* = 0.126) than DT (*M* = 3.01, *SE* = 0.129), *t*(22) = 3.773, *p* = 0.001, Cohen's d = 1.61 (Figure 7).

We did not observe any significant difference in perspective-taking task performance between the two display conditions, *t*(22) = 0.416, *p* = 0.682. Exploration in either of the display modes also did not differ in sense of presence they created, either within the display mode, *t*(22) = 1.073, *p* = 0.295, or outside, *t*(22) = 0.283, *p* = 0.780 (Figure 7).

## Discussion

The current study investigated the effect non-ambulatory HMD VR in comparison to DT VR on spatial learning from navigating a virtual environment, and whether the VR related disruptive effects, such as VRISE and increased workload, can account for those differences. We found an advantage of DT over HMD display mode for learning spatial relations for two out of four spatial components of the navigated virtual environment. These two components were topology of junctions in a given street network, and cyclic order of street segments and object around a junction. Recall of spatial components related to topology of street segments, and object associations was same regardless of whether the virtual environment was navigated using DT or HMD VR. These results highlight that the differential effect of DT vs. HMD VR depends on the nature of cognitive and functional behavior that the user employs. We also found that although both VRISE and subjective workload was higher in HMD than DT condition, neither of these factors accounted for the obtained differences in learning the four spatial components across the two display conditions. One reason for this could be motion-sickness or workload in both the display conditions was overall very low. Participants explored the VE longer under DT than HMD condition, but no difference was obtained in the sense of presence and perspective-taking. We interpret our results as participants performed better or equally well on recalling details of different spatial components of a navigated virtual environment when the navigation was done using a DT vs. HMD VR, regardless of the effect of disruptive factors such as VRISE or workload.

The recent proliferation in VR technology seems to be very promising for both the IT and entertainment industry, as well as a research tool that provides experimenters with greater control over experimental manipulations and with a host of possibilities for creating stimuli. However, it is not clear how beneficial is the higher degree of immersion and visual fidelity of VR systems for performance in a virtual environment, and how disruptive can some of the VR induced sickness and effects can be. Our findings addresses both these issues.

Previous studies have shown that participants do worse under HMD condition as compared to other display conditions, such as DT, 2D, or stereoscopic 3D conditions (Manrique, 1998; Santos et al., 2009; McMahan et al., 2012; Stevens et al., 2015; Roettl and Terlutter, 2018). However, other studies have also reported similar effect or selective advantage (Parmar et al., 2016) or better performance using HMD than DT VR (Murcia-López and Steed, 2016; Parmar et al., 2016). These studies differ in terms of how they control the same variable across experiments, and one particular issue that we were interested in addressing in the current study, was to tease apart the effect of HMD's interactional fidelity from visual fidelity in making HMD advantageous over DT. Previous studies have shown that idiothetic information helps in navigation and spatial learning (Chrastil and Warren, 2012, 2013). Therefore, HMD when used with walking locomotion interface would benefit users' performance. Restricting ambulatory locomotion in HMD would help us record an unmitigated effect of high immersiveness and visual fidelity on performance in VR. But it could be possible that non-ambulatory HMD may result in disruptive effects of VRISE and increased task effort, and counteract the overall benefit of higher immersiveness that HMD has over DT, which may result in performance in HMD to be same, if not worse, than in DT. For a better understanding of how HMD affects human spatial task performance, we need to know how different components of HMD VR or features that are sometimes included with it, such as walking locomotion, affect users' behavior. Our results show that when we restrict podokinetic locomotion in HMD, the performance in an immersive HMD is actually equal, if not worse, than DT VR. Note that this effect is obtained after controlling for the disruptive effects of immersiveness, which was higher in HMD than DT condition. This indicates that the level of visual fidelity and immersiveness that DT provides is at least equally efficient, if not better, for topological spatial learning task in comparison to the higher level of visual fidelity and immersiveness of HMD. Our results provide support to previous findings that show better or equal performance using DT as compared to HMD VR (Manrique, 1998; Santos et al., 2009; McMahan et al., 2012; Stevens et al., 2015; Parmar et al., 2016; Roettl and Terlutter, 2018).

Comparing our results with those of Murcia-López and Steed (2016) that reported better spatial learning performance in HMD with locomotion than DT, we can draw the conclusion that it is the idiothetic information coming from self-reference locomotion that seems to be the primary reason for better performance in HMD than DT. If we minimize that information by restricting walking locomotion, the benefit of HMD over DT is lost and in fact the effect might be reversed.

Our findings could also be interpreted in the light of previous findings that show positive effects of ego-centric locomotion-based perceptual-motor information on navigation. Recently, van der Ham et al. (2015) investigated the role of physical involvement in acquiring spatial knowledge. In this study, participants explored real, hybrid (virtual + real), and virtual environment. In real and hybrid conditions, participants used walking for exploring the environment, however in virtual condition, participants used keyboard and mouse to explore the virtual environment. They found real environment navigation was best in acquiring survey knowledge. The performance on hybrid condition did not differ significantly from the real environment. However, the virtual environment showed the poorest performance on survey knowledge. Studies by Chrastil and Warren (2013) showed similar effect when participants used walking compared to keyboard action for virtual locomotion during HMD VR way-finding task. These findings indicate the importance of perceptuo-motor coordination generated by ego-centric locomotion during navigation in HMD VR condition. Since locomotion-generated ego-centric visual feedback matches the real navigation experience, it creates higher fidelity in HMD than DT VR which positively affects spatial learning and makes it similar to learning in the real environment (Chrastil and Warren, 2013, 2015; van der Ham et al., 2015; Murcia-López and Steed, 2016). However, the current study provides only indirect evidence for the role of ambulatory information in virtual spatial learning. Even though the current design is helpful in looking at the effect of non-ambulatory HMD, future work should directly compare the effect of ambulatory vs. non-ambulatory HMD on users' performance.

The current result suggests that when the virtual environment lacks the idiothetic cues generated by walking, then 2D display mode appears to be comparable, if not better, source of spatial learning with respect HMD VR. The advantage of DT over HMD could be because of higher familiarity, comparatively lesser sensory conflict, and cognitive demand during exploration, which would leave more mental resources available to learn the spatial layout (Fenske and Eastwood, 2003; Green and Bavelier, 2006; Srivastava et al., 2010; Terlutter et al., 2016; Yim et al., 2017; Roettl and Terlutter, 2018). Similarly, studies comparing the effect of 2D, with either 3D, 4D, and/or HMD VR on spatial memory while playing video game using X-box game controller, showed better recall and recognition of relevant objects in the environment with 2D compared to other display modes (Terlutter et al., 2016; Yim et al., 2017; Roettl and Terlutter, 2018). The authors of these studies interpreted the finding as supporting the higher usability of 2D display mode because of lower sensory-motor engagement and lower perceptual and cognitive load.

Although, DT seems to favor the acquisition of spatial relations, it took significantly longer time to complete the virtual exploration than HMD VE. It could be due to either more time was required to learn the environment because of 2D visual effect, or ease of exploration due to lesser sensory conflict leading to reduced visually induced motion sickness. A plausible explanation on why DT VR exploration took longer time than HMD VR, could be due to the difference between visual feedback acquired during exploration task in both the display modes. The head rotation in HMD enabled participant to naturally move their head right/left while moving around, whereas in DT, participants were required to halt at a particular place in VE to explore their reference point in the VE. This could result in more navigation time in the DT vs. HMD condition. However, this might have resulted in sensory-motor conflict because of lack of walking information, and might have created a discomfort in exploring the HMD VR. Studies evaluating HMD VR sickness showed that it is a multifactorial problem and could be caused by any of the VR technical features related to the field of view, optical distortion, flicker, refresh rate, resolution, latency and poor tracking of visuo-motor feedback, slow update rate, etc. (Sharples et al., 2008; Toet et al., 2008; Jerald, 2016; Lu, 2016). The high compared to low realism graphics causes more visual flow and stronger sensory conflict, especially in case of mismatch between visual and vestibular ego-centric feedbacks, which leads to visually induced motion sickness (Toet et al., 2008; Keshavarz et al., 2015; Jerald, 2016; Lu, 2016; Yim et al., 2017; Ng et al., 2018). This could indirectly support our result that participants spent more navigation time in DT than HMD display as they experienced less VRISE and task effort in DT than HMD condition. The current study is limited in refuting the alternative hypothesis. Future studies with exploration strategies might be helpful in testing the two rival positions.

As hypothesized that restricting walking might result in higher discomfort and effort, we observed comparatively higher VRISE and task effort in HMD compared to DT condition. This result alludes to the findings from Chrastil and Warren (2013) study, in which they observed comparatively lesser nausea rating with walking HMD condition than otherwise. However, we defer from strongly making that claim as the overall VRISE and workload perception was low. Future studies should examine if our current survey measures are sensitive enough to measure disruptive effects of VR technologies.

Studies comparing the DT and HMD VR have also shown a higher sense of presence with HMD VR than DT VR (Terlutter et al., 2016; Yim et al., 2017; Roettl and Terlutter, 2018). However, the current result did not show any difference between the display modes. It could be due to the low interaction fidelity of the display conditions, or sensitivity of the measure of presence.

There were limitations in our study. We let the participant decide how long they wanted to explore the VE within a maximum allowed duration. We found that participants spent more time in DT vs. HMD condition and this could explain the results of similar or better spatial learning in DT vs. HMD, and would therefore lead to confounding in our study. Our decision to cap the maximum time for virtual navigation was inspired by previous studies (Boonsuk et al., 2012; Murcia-López and Steed, 2016; Raviptai and Sahu, 2017; Sahu et al., 2017). Time spent on the task may have an influence on acquiring spatial information. We were interested in finding how the recall performance on the map-sketching task would differ for the two conditions, after the users had navigated the VE for a sufficient duration so that they had subjectively felt that they could later sketch the map of the VE. Since the participation session had to end, we decided a maximum duration based on previous studies. We thought our manipulation to allow participants to decide the navigation time with an upper time limit had more ecological validity with respect to how users would naturally use the two interfaces. To have participants explore a VE for a specific duration after they have already felt that they have sufficiently explored it, could induce boredom and practice effect. The amount of time volitionally spent on a task may itself be a result of nature of the task, that is, the set-up or the virtual environment of DT vs. HMD could be such that participants naturally spend more time in DT vs. HMD. One reason for it could be that participants found DT to be more familiar and easier to interact with than HMD. Another reason could be that VRISE and effort could be more in HMD than DT, or natural head movement in HMD as compared to computer peripheral controls in DT could cost more time in the latter. These are the reasons we left it on the participants to decide if they had felt that they navigated the VE sufficiently enough so that later they can draw a map sketch of the virtual layout.

Our participants were seated at 80 cm from the computer monitor in the DT condition. This created a different FOV for the two display conditions. In both the conditions, the virtual cameras projected 110° FOV of the virtual environment on the corresponding display modes. In HMD condition, the virtual camera FOV matched the HMD FOV. However, in DT condition, the virtual camera was not aligned with the viewers' 110° FOV. We acknowledge that this difference in FOV may explain part of our results but it is not clear to us if it would necessarily make spatial learning worse in HMD as compared to DT, and what effect it would have for a broader-use implementation of our findings using the two VR technologies.

## Conclusion

Our study shows that high visual fidelity of HMD VR in absence of high interaction fidelity makes it as good as if not worse than DT VR for spatial learning, and this effect depends on the nature of the studied behavior. Participants in DT vs. HDM condition were better in recalling two types of spatial components (junction & cyclic order) of the navigated virtual space, and performed equally well on other two components (street segments & object associations). The limitation in replicating Murcia-López and Steed (2016)'s finding of better spatial learning in HMD vs. DT is explained in terms of lack of ambulatory feedback generated by physical walking, which might have played a crucial role in favoring HMD VR (Chrastil and Warren, 2013, 2015; van der Ham et al., 2015). The current results extend the previous findings of worse or similar performance in a virtual setting in which the interaction fidelity was low during exploration (Santos et al., 2009; McMahan et al., 2012; Stevens et al., 2015; Parmar et al., 2016; Roettl and Terlutter, 2018). One reason why HMD VR in our study was not more helpful than DT VR for spatial learning could be that it caused more motion sickness and involved more effort. These results highlight the comparative importance of different components that constitute immersive technology (e.g., HMD VR), the usability of less immersive technology (e.g., DT VR), and underscores the importance of idiothetic information in virtual exploration and spatial learning. The current study will be helpful in designing VR settings for military reconnaissance task, surveillance task, or search and rescue operations, which requiring remote operators to acquire spatial knowledge about environments with restricted or high-risk locomotive accessibility. Our findings are qualified by the limitations that there was overall very low disruptive VR effects in the HMD (and DT) condition, the FOV was not exactly matched for the two display conditions, and most of the participants were male.

## Ethics Statement

This study was carried out in accordance with the recommendations of Association for Psychological Sciences ethics guidelines, Institute Human Ethics Review Board committee, IIIT-Hyderabad with written informed consent from all subjects. All subjects gave written informed consent in accordance with the Declaration of Helsinki. The protocol was approved by the Institute Review Board committee, IIIT-Hyderabad.

## Author Contributions

PS conceptualized the idea, designed the virtual environment layout, designed and planned the experiment, conducted analyses, and wrote the paper. AR played an equally critical role in conceptual discussion, experimental planning, and did major share of the data analysis and paper writing. PV and SS played an important role in writing UNITY 3D code for the virtual environment, worked on synchronizing virtual reality and DT setup and their respective data logging, survey design, data collection, and conducted a coding of the data for inferential statistics. SC helped in developing the idea regarding spatial knowledge required in military reconnaissance situation.

### Conflict of Interest Statement

The authors declare that the research was conducted in the absence of any commercial or financial relationships that could be construed as a potential conflict of interest.
